# *Ab initio* simulations of α- and β-ammonium carbamate (NH_4_·NH_2_CO_2_), and the thermal expansivity of deuterated α-ammonium carbamate from 4.2 to 180 K by neutron powder diffraction

**DOI:** 10.1107/S2052520622002645

**Published:** 2022-04-30

**Authors:** Christopher M. Howard, Ian G. Wood, Kevin S. Knight, A. Dominic Fortes

**Affiliations:** a Bayerisches Geoinstitut (BGI), University of Bayreuth, 95447 Bayreuth, Germany; b Department of Earth Sciences, University College London, Gower Street, London WC1E 6BT, United Kingdom; c ISIS Facility, Rutherford Appleton Laboratory, Harwell Science and Innovation Campus, Didcot, Oxfordshire OX11 0QX, United Kingdom

**Keywords:** powder diffraction, thermal expansion, DFT, neutron diffraction, negative linear compressibility, ammonium carbamate

## Abstract

Experimental and computational studies of ammonium carbamate have been carried out, with the objective of studying the elastic anisotropy of the framework manifested in (i) the thermal expansion and (ii) the compressibility; furthermore, the relative thermodynamic stability of the two known polymorphs has been evaluated computationally. Using high-resolution neutron powder diffraction data, the crystal structure of α-ammonium carbamate (ND_4_·ND_2_CO_2_) has been refined [space group *Pbca*, *Z* = 8, with *a* = 17.05189 (15), *b* = 6.43531 (7), *c* = 6.68093 (7) Å and *V* = 733.126 (9) Å^3^ at 4.2 K] and the thermal expansivity of α-ammonium carbamate has been measured over the temperature range 4.2–180 K. The expansivity shows a high degree of anisotropy, with the *b* axis most expandable. The *ab initio* com­putational studies were carried out on the α- and β-polymorphs of ammonium carbamate using density functional theory. Fitting equations of state to the *P*(*V*) points of the simulations (run athermally) gave the following values: *V*_0_ = 744 (2) Å^3^ and bulk modulus *K*_0_ = 16.5 (4) GPa for the α-polymorph, and *V*_0_ = 713.6 (5) Å^3^ and *K*_0_ = 24.4 (4) GPa for the β-polymorph. The simulations show good agreement with the thermoelastic behaviour of α-ammonium carbamate. Both phases show a high-degree of anisotropy; in particular, α-ammonium carbamate shows unusual compressive behaviour, being determined to have negative linear compressibility (NLC) along its *a* axis above 5 GPa. The thermodynamically stable phase at ambient pressure is the α-polymorph, with a calculated enthalpy difference with respect to the β-polymorph of 0.399 kJ mol^−1^; a transition to the β-polymorph could occur at ∼0.4 GPa.

## Abstract 2

The results of *ab initio* simulations carried out using density functional theory on two polymorphs of ammonium carbamate are reported. Also reported is a refined crystal structure of deuterated α-ammonium carbamate from high-resolution neutron powder diffraction data, with thermal expansion measurements from 4.2 to 180 K.

## 1. Introduction

**Figure 1:**
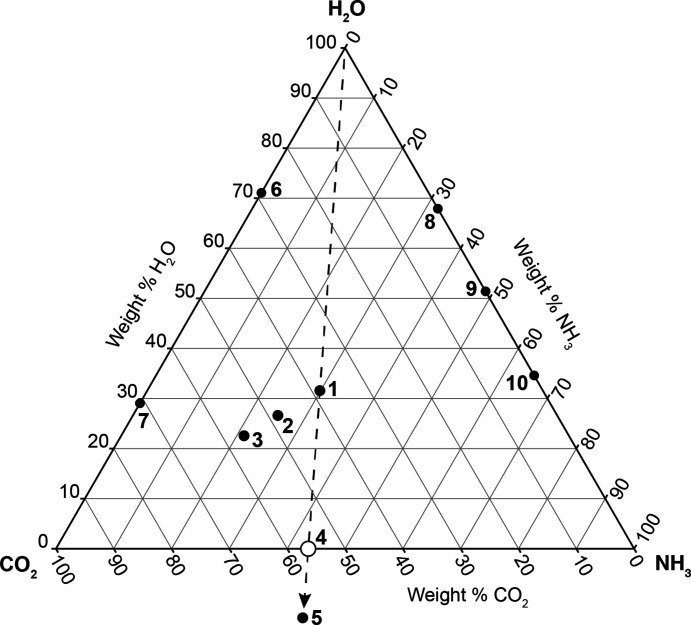
The ternary system of NH_3_ + CO_2_ ± H_2_O. The compositions to form in this system are: (**1**) ammonium carbonate monohydrate; (**2**) ammonium sesquicarbonate monohydrate; (**3**) ammonium bicarbonate; (**4**) α- and β-ammonium carbamate (open circle); (**5**) urea; (**6**) CO_2_ clathrate hydrate; (**7**) solid carbonic acid; (**8**) ammonia dihydrate; (**9**) ammonia monohydrate; (**10**) ammonia hemihydrate.

With recent robotic missions to icy planetary bodies in our solar system, most notably the flybys of Pluto, Charon and Arrokoth by the New Horizons spacecraft, and of Ceres by the Dawn spacecraft, the importance of characterizing ‘ices’ composed of primordial mixtures of ammonia, carbon dioxide and water has become more urgent. Although long suspected to be present in the outer solar system (Kargel, 1992 ▸), solids in the ternary NH_3_ + CO_2_ ± H_2_O system (Fig. 1 ▸), which includes two phases of ammonium carbamate (NH_4_·NH_2_CO_2_), am­monium carbonate monohydrate [(NH_4_)_2_CO_3_·H_2_O], ammonium sesquicarbonate monohydrate {(NH_4_)_4_[H_2_(CO_3_)_3_]·H_2_O}, ammonium bicarbonate [(NH_4_)_2_HCO_3_] and the water-deficient compound urea [CO(NH_2_)_2_], appear increasingly likely to occur as ‘rock-forming’ minerals.

Pluto has extensive deposits of N_2_, CH_4_ and CO ices across its surface in various mixtures (Protopapa *et al.*, 2017 ▸; Schmitt *et al.*, 2017 ▸; Moore *et al.*, 2016 ▸) and a thin N_2_-dominated atmosphere (Gladstone & Young, 2019 ▸), with both Pluto and its moon Charon manifesting the spectroscopic signature of ammoniated material (Dalle Ore *et al.*, 2018 ▸, 2019 ▸; Grundy *et al.*, 2016 ▸). The dwarf planet Ceres also manifests spectroscopic signatures of ammoniated species, in the form of phyllo­silicates (Ammannito *et al.*, 2016 ▸; Raponi *et al.*, 2019 ▸), which are seen in the regions with high albedo, in particular in the complex crater Occator (De Sanctis *et al.*, 2015 ▸, 2016 ▸), suggesting an NH_4_-rich inter­ior. The Kuiper belt object Arrokoth has abundant CH_3_OH on the surface (Stern, 2019 ▸), with other potential ices, including H_2_O, NH_3_, H_2_CO and CO_2_, in trace amounts (Grundy *et al.*, 2020 ▸). In other words, all the constituents necessary to make ammonium carbonates (and related substances) are present and so are likely to form these materials wherever partial melts of ammonia–water are exposed to solid or gaseous CO_2_.

In the same context, ammonium carbonates may have some astrobiological relevance since removal of water leads – *via* ammonium carbamate – to urea. It is known from laboratory analogue experiments that the organic mol­ecules produced photochemically in the atmosphere of Saturn’s giant satellite Titan may be hydrolysed in aqueous ammonia to form both urea and amino acids (Poch *et al.*, 2012 ▸). Further hydrolysis of urea would be expected to form ammonium carbonates; on Earth, this process is mediated in soils with the aid of bacterial urease, whereafter the carbonate breaks down to ammonia and water. Meteorite impacts into Titan’s surface would provide the requisite liquid (as impact melt) to hydrolyse any solid organics (Artemieva & Lunine, 2003 ▸) and both urea and ammonium carbamate/carbonate may be substantial by-products, persisting on geological timescales at the surface temperature of 95 K.

Not only are ammonium carbonates relevant to the outer solar system, but they also play a role in industry here on Earth; ammonia–water solutions may be used to capture CO_2_ from flue gases, deposited in solid form as ammonium carbonates (Han *et al.*, 2013 ▸). Furthermore, there has been some inter­est in using ammonium carbonates for hydrogen storage in fuel cells (Lan *et al.*, 2012 ▸).

There has nevertheless been comparatively little inter­est in studying the structures and properties of these compounds; indeed, the structure of ammonium carbonate monohydrate was determined comparatively recently (Fortes *et al.*, 2014 ▸), and the structure of the sesquicarbonate was determined in 2003 (Margraf *et al.*, 2003 ▸). Furthermore, the identity of a second (β) phase of ammonium carbamate was first reported in 2007 (Kuhn *et al.*, 2007 ▸), while the only published crystal structure of α-ammonium carbamate was that determined in 1973 by single-crystal X-ray diffraction (Adams & Small, 1973 ▸). Both ammonium carbamate phases are ortho­rhom­bic: α-ammonium carbamate has the space group *Pbca*, with *Z* = 8, while β-ammonium carbamate has the space group *Ibam*, with *Z* = 8.

It is surprising that ammonium carbamate has apparently been of little inter­est, given the similarity of the carbamate ion to the primary amide group; previous work on ammonium carbamate has mainly focused on the thermodynamics and kinetics, typically related to the formation of urea, rather than crystallographic studies. Whilst urea has been extensively studied, the same is not true of the carbonate/carbamate family of compounds.

The goals of this study were to calculate the bulk properties of both phases of ammonium carbamate as a function of pressure, preparatory to the development of a planetary model. Without knowing how something as fundamental as the density varies with pressure and temperature, as well as high-pressure polymorphism, the development of accurate structural and evolutionary models of icy planetary inter­iors is severely limited. To determine the necessary physical properties includes calculating the bulk elastic properties as a function of pressure using density functional theory (DFT); in particular, how the different arrangement of structural units and hydrogen bonds affects the bulk modulus of the two polymorphs.

An opportunity to test the anisotropy of the α-polymorph arose during an attempt to measure the thermal expansion of ammonium carbonate monohydrate. During a synthesis of that product, ammonium carbamate was formed instead, and neutron powder diffraction data were collected as a function of temperature. The thermal expansion derived from these data provides a useful measurement of the structural anisotropy for comparison with the calculations.

Here, therefore, we present the first *ab initio* calculations to determine the high-pressure behaviour of both α- and β-am­monium carbamate; the first neutron powder diffraction data of α-ammonium carbamate, thereby providing the first accurate H-atom positions to allow characterization of the hydro­gen bonding in the crystal; and thermal expansivity measurements from 4.2 to 180 K. These results begin to bridge the current gap in our knowledge of the behaviour of ammonium carbonates under conditions related to the icy moons of the outer solar system.

## 2. Experimental

Time-of-flight neutron powder diffraction data were collected using the ‘High Resolution Powder Diffractometer’ (HRPD) at the STFC ISIS neutron spallation source of the Rutherford–Appleton Laboratory, UK. The HRPD instrument was chosen due to its high resolution (Δ*d*/*d* ≃ 8 × 10^−4^ in the backscattering bank), allowing the highest precision in refined unit-cell parameters and accurate partitioning of intensity in well-resolved high-*Q* Bragg peaks.

### 2.1. Sample preparation

Ammonium carbamate was formed serendipitously in the process of attempting to synthesize deuterated ammonium carbonate monohydrate, during which a 28 wt% ND_3_ solution was exposed to CO_2_ from sublimating dry ice pellets, forming a slurry of white material. Consequently, the specimen contained excess water in the form of water ice, which also acted as a reservoir for small amounts of excess ammonium. The resulting white compound was stored in a freezer. The sample was taken out of the freezer and allowed to warm and soften, after which the partially molten slurry was transferred into a cryomortar under liquid nitro­gen and ground to a fine powder using a similarly cooled pestle. Once fully ground, the material was loaded into an aluminium-framed slab-geometry can of inner dimensions 23 × 18 × 15 mm (h × w × d) relative to the incident neutron beam, also precooled in liquid N_2_. Vanadium foil windows were indium-sealed to the front and back faces of the slab can, with the exposed components on the incident-beam side being masked with Gd foil. The sample holder was wired with an RhFe resistance thermometer and a 30 mm cartridge heater, after which, it was immersed in liquid nitro­gen for transfer into a He cryostat operating at 100 K. Once oriented in the beam, the cryostat temperature was reduced to 4.2 K for measurements to commence.

### 2.2. Data acquisition

Time-of-flight data on HRPD are measured routinely in a range of 100 ms bandwidth ‘windows’ where disc choppers act as bandwidth selectors, these typically being 30–130 or 100–200 ms. In the instrument’s backscattering detector bank (2θ = 154–176°), these time-of-flight ranges provide access to *d*-spacings in the ranges 0.65–2.60 (30–130 ms) and 2.15–4.00 Å (100–200 ms) at the highest resolution. Inter­mediate-resolution data are simultaneously measured in the 90° detector banks (0.85–3.90 Å in 30–130 ms and 2.82–6.00 Å in 100–200 ms). Since HRPD has an essentially *Q*-independent resolution function, we can obtain high-precision unit-cell parameters from data measured only in the 100–200 ms win­dow whilst avoiding peak overlap from the additional peaks at shorter *d*-spacings. For accurate structure refinement, how­ever, it is more desirable to measure the shorter *d*-spacing peaks in the 30–130 ms window.

Consequently, we collected a low-noise structural data set at 4.2 K in the 30–130 ms window, counting for 6.5 h (corresponding to an integrated proton beam current of 250 µA h) and then measured on warming using only the 100–200 ms window. These thermal expansion data were collected in 5 K increments from 10–150 K and (due to time constraints) in 10 K increments up to 180 K. Counting times were 15 min, cor­responding to an integrated proton beam current of 15 µA h per step. The diffraction data were focussed, nor­malized to the incident beam spectrum and corrected for detector efficiency by reference to a vanadium standard using *Mantid* (Arnold *et al.*, 2014 ▸) and then exported in a format suitable for analysis with *GSAS*/*Expgui* (Larson & Von Dreele, 2004 ▸; Toby, 2001 ▸).

### 2.3. Structural refinement

**Figure 2:**
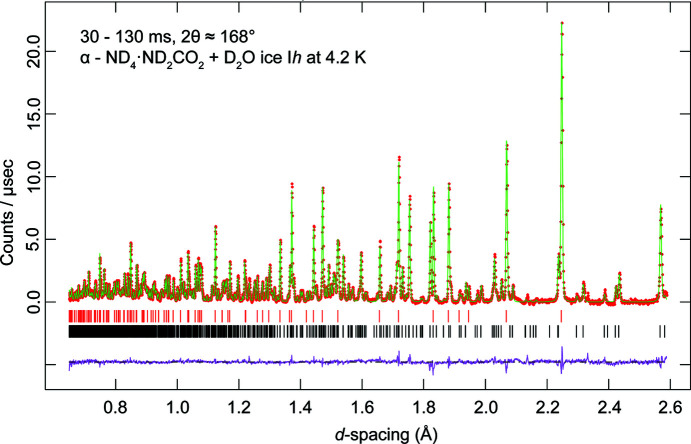
Neutron powder diffraction pattern acquired at 4.2 K in the highest resolution backscattering banks of HRPD. Red symbols represent the measured data, green lines the result of Rietveld refinement, pink lines the difference profile and the tick marks the expected positions of each Bragg reflection for ND_4_·ND_2_CO_2_ (black) and D_2_O ice I*h* (red).

In the 4.2 K neutron powder diffraction dataset, two phases were identified by inspection of the peak positions – deuterated water ice and α-ammonium carbamate. The Rietveld method was used to refine the structure of D_2_O ice I*h* and α-ammonium carbamate, starting from the previously published atomic coordinates based upon the single-crystal X-ray investigation of Adams & Small (1973 ▸). In this refinement, the unit-cell parameters, scale factor, peak-profile coefficients (*GSAS* profile function #3) and a twelve-term Chebyshev polynomial background were allowed to vary and the atomic coordinates and isotropic displacement parameters (*U*_iso_) were refined independently, without restraints. It was found necessary to refine a wavelength-dependent absorption/reflectivity correction (*GSAS* model #1). The final fit to the data is excellent, the *R*_wp_ value being better than 2%; a graphical depiction of the fit to the neutron powder data is given in Fig. 2 ▸. The refined structural parameters are given in the crystallographic information file (CIF) in the supporting information.

**Figure 3:**
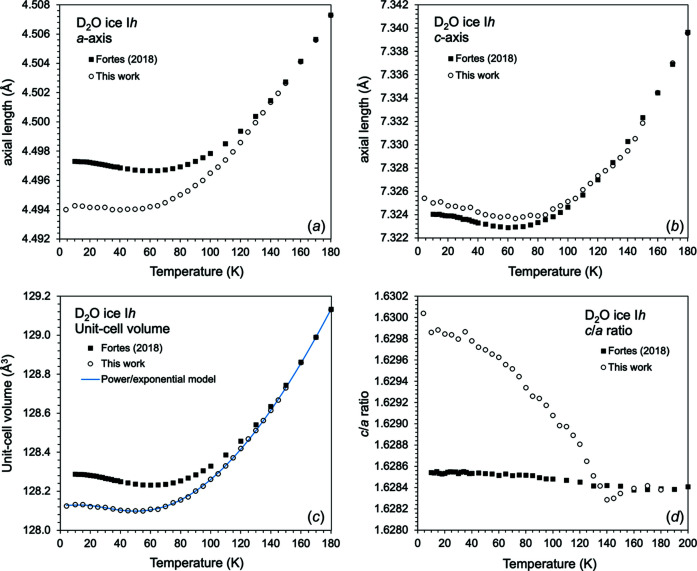
(*a*)/(*b*) Refined unit-cell parameters, (*c*) unit-cell volume and (*d*) *c*/*a* ratio of D_2_O ice I*h* compared to literature values. Solid symbols are from Le Bail refinements at each temperature step and black symbols are from Fortes (2018 ▸). There is a clear change in thermal expansion behaviour – principally of the *a* axis – below *ca* 135 K.

At each temperature step, the unit-cell parameters of both phases were determined using the Le Bail method (with initial intensities based on the *GSAS F*_calc_ method); in addition to the unit-cell parameters, the peak profile parameters and the background parameters were allowed to vary. Consideration of the axial ratio of the D_2_O ice I*h* present in the sample (see Fig. 3 ▸ and §4.1) indicated that, at 180 K, it was sufficiently pure to allow its use as an inter­nal standard. To ensure uniformity with previous work, the unit-cell parameters of D_2_O at 180 K were therefore fixed to those measured in a previous experiment with a silicon standard (Fortes, 2018 ▸), and the diffrac­tometer constants subsequently refined.

## 3. Computational methods

To provide further information for planetary models, such as high-pressure behaviour, and to provide an accurate structure for β-ammonium carbamate, both structures of ammonium car­bamate were input into *CASTEP* (Clark *et al.*, 2005 ▸). Static density functional theory (DFT) calculations were then carried out using the PBE pseudopotential (Perdew *et al.*, 1996 ▸) with the Tkatchenko–Scheffler dispersion correction (Tkatchenko & Scheffler, 2009 ▸). The importance of dispersion corrections for the study of elastic properties and anisotropy in planetary ices was demonstrated recently by Meusburger *et al.* (2021 ▸). For the work reported here, we had found that geometry optimizations completed without dispersion cor­rections led to disagreements of ∼10% in the unit-cell parameters, these being reduced to 1–3% with the use of dis­persion corrections. Similarly, the estimated phase-transition pressures reported later were reduced from ∼2 to ∼0.4 GPa.

Convergence tests were carried out to optimize the *k*-point sampling of the Brillouin zone within the Monkhorst–Pack scheme (Monkhorst & Pack, 1976 ▸) and the kinetic energy cut-off of the plane-wave basis set. For α-ammonium carbamate, a converged grid of 2 × 6 × 5 (∼0.03 Å^−1^ reciprocal lattice spacing), with an energy cut-off of 1000 eV, yielded a total energy convergence better than 10^−3^ eV. For β-ammonium carbamate, a *k*-point mesh of 2 × 2 × 3 (∼0.05 Å^−1^ reciprocal lattice spacing), with an energy cut-off of 980 eV, yielded a total energy convergence better than 10^−3^ eV.

A series of calculations were then performed in which the ions were allowed to move according to the Hellman–Feynman forces with the symmetry maintained, and the unit-cell shape was allowed to vary; the initial structural parameters of α-ammonium carbamate were those obtained from our neutron powder diffraction data at 4.2 K and of β-ammonium carbamate were taken from Kuhn *et al.* (2007 ▸).

## 4. Results and discussion

### 4.1. Behaviour of D_2_O ice I*h* upon warming

When comparing D_2_O ice I*h* unit-cell parameters to literature values, there is a significant difference in the ice unit-cell parameters compared with pure ice at low temperatures (Fig. 3 ▸). Although the unit-cell volumes at 180 K agree well, there is a growing divergence at lower temperatures, such that the unit-cell volume is smaller than that of pure D_2_O ice I*h* at 10 K by 0.11% and the transition to negative volumetric thermal expansion is shifted to a lower temperature. Fur­ther­more, the deviation is associated almost entirely with the behaviour of the crystal’s *a* axis; this is most apparent from a plot of the *c*/*a* ratio against temperature (Fig. 3 ▸). From 180 to 135 K, the *c*/*a* ratio is in excellent agreement with both the absolute values and the temperature dependence found in pure D_2_O (Fortes, 2018 ▸); below 135 K, the *c*/*a* ratio increases systematically.

The dependence of the *c*/*a* ratio in ice I*h* on both thermal history and chemical doping was described by Fortes (2019 ▸), who attributed the observed effects to changes in partial order in the crystal. We therefore propose that small concentrations of ammonium ions present in our D_2_O ice I*h* sample effects similar very subtle structural changes that drive the *c*/*a* ratio to adopt a nearly ideal hexa­gonal close-packed value (1.630).

**Figure 4:**
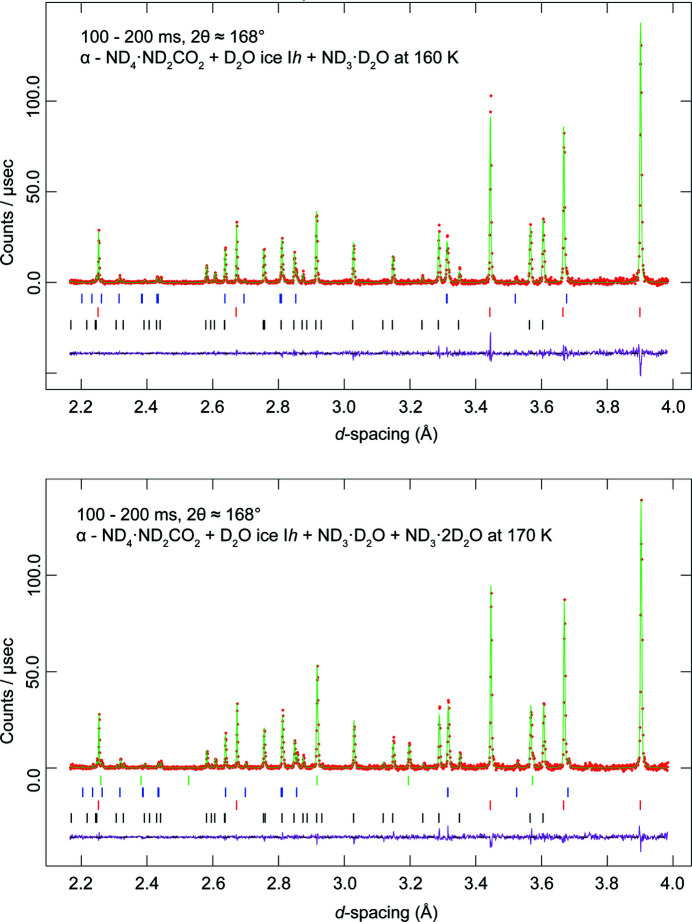
Neutron powder diffraction patterns acquired at 160 K (top) and 170 K (bottom) in the highest resolution backscattering banks of HRPD. Red symbols represent the measured data, green lines the result of Rietveld refinement, pink lines the difference profile and the tick marks the expected positions of each Bragg reflection for: (*a*) ND_4_·ND_2_CO_2_ (black); (*b*) D_2_O ice I*h* (red); (*c*) ND_3_·D_2_O (blue); (*d*) ND_3_·2D_2_O (green). At 160 K, peaks of ND_3_·D_2_O appear, and at 170 K, peaks of ND_3_·2D_2_O also appear; by 180 K, these peaks have disappeared.

In addition to the likely very small qu­anti­ties of ammonium ions trapped in the crystal structure of ice I*h*, it appears that there was also sufficient excess ammonia present – likely as an amorphous aqueous solid in pores and on grain boundaries – to form ammonia hydrates. These were seen in the diffraction patterns during warming of the sample, with the first impurity phase appearing at 160 K. Peaks from this phase became larger at 170 K and were joined by peaks from another accessory phase (Fig. 4 ▸). During the final measurement at 180 K, all impurity peaks had disappeared completely.

We matched the peaks from both accessory phases; the first to appear was ammonia monohydrate (ND_3_·D_2_O) and the second was ammonia dihydrate (ND_3_·2D_2_O). Since these are both known to melt between 170 and 180 K, it is not surprising that they are absent from the 180 K diffraction data.

The presence of excess water in the form of ice meant we were obliged to keep the sample below the melting point of water. The addition of even small amounts of water from moisture in the atmosphere to ammonium carbamate is enough to slowly form ammonium bicarbonate. With the presence of ammonium ions in the sample, eventually melting and forming ammonium hydrates, the melting point of water would be significantly reduced, and we could not be certain as to when the sample would start to decompose, so it was decided to halt the experiment at 180 K to be certain the sample did not melt.

### 4.2. α-Ammonium carbamate

#### 4.2.1. Structure and bonding

**Figure 5:**
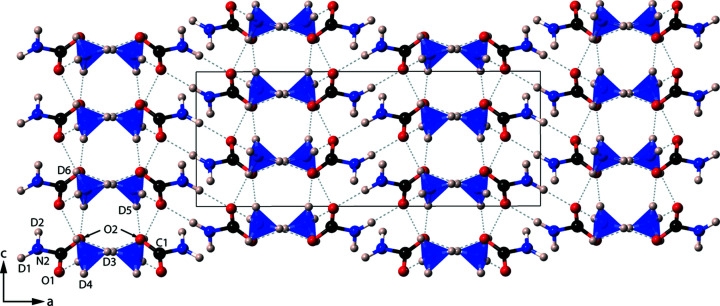
The structure of α-ammonium carbamate, viewed parallel to the *b* axis. The grey dashed lines are H(D)⋯O hydrogen bonds, and the solid black line is the unit cell.

**Figure 6:**
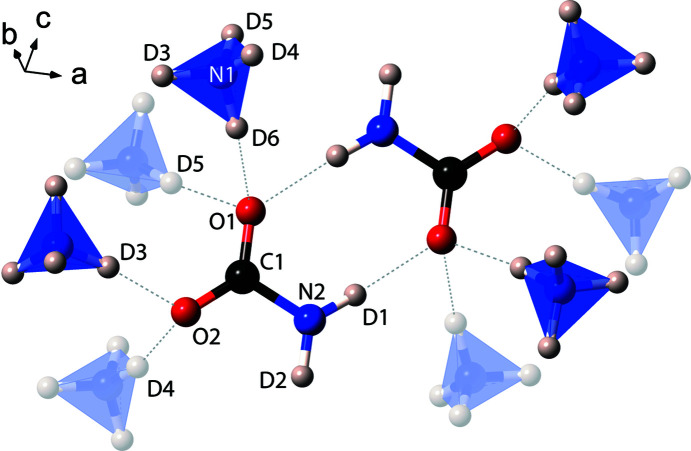
The 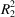 (8) carbamate ionic dimers seen in the structure of α-ammonium carbamate (ND_4_·ND_2_CO_2_). Faded tetra­hedra are below the plane of the centrosymmetric ring structure and the dashed lines represent H(D) bonds.

The structure consists of ND_4_^+^ tetra­hedra and planar ND_2_CO_2_^−^ anions linked by moderately strong hydrogen bonds [H(D)⋯O in the range 1.690–1.949 Å]. Pairs of carbamate ions run parallel to the *b* axis, which are linked *via* ammonium tetra­hedra (Fig. 5 ▸). Hydrogen bonding between centrosymmetric pairs of carbamate ions creates an 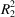 (8) ring motif, measuring 4.286 Å, between centrosymmetric pairs of C atoms; these rings are typically seen in primary amide structures, with amide mol­ecules, *R*-CONH_2_, forming the rings (Fig. 6 ▸).

There are many different possible arrangements of these rings within a crystal structure, including various ring, tape, and sheet-like motifs (Leiserowitz & Hagler, 1983 ▸). In ammonium carbamate, layers of these hexa­gonal rings are alternately arranged perpendicular to one another, known as a herringbone motif arrangement. Indeed, the space group of ammonium carbamate, *Pbca*, can generate an inter­layer herringbone motif between the terminal atoms of the amide residue groups, *R*, which lie on the outside of the bilayer, due to the *b*-glide plane (Leiserowitz & Hagler, 1983 ▸). The form of the ammonium carbamate motifs in α-ammonium carbamate is therefore similar to the shallow glide motif in primary amide structures.

In the shallow glide motif, the spacing between layers of the same orientation is typically 3.4 Å; in ammonium carbamate, this spacing is almost doubled in length, at 6.436 or 6.681 Å (equivalent to the *b* and *c* axes, respectively), due to the inter­layer motif acting as an additional layer. Three C atoms of separate hexa­gonal ring structures usually have a separation of ∼10 Å, but here the distance is 9.27 Å; this is accommodated by the tilt angle of the ring structures being ∼68° rather than a typical 61° (Leiserowitz & Hagler, 1983 ▸).

In terms of bonds and bond lengths (see Table 1 ▸), the length of the N2—D1 bond is longer than the N2—D2 bond, due to the D1 atom donating a hydrogen bond [forming part of the 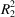 (8) H(D)-bonded motif]. The N2—D2 bond was suggested not to be involved in a hydrogen bond by Adams & Small (1973 ▸); it appears that this H(D) atom may be sharing an inter­action with the O1 atom, although the (H)D2⋯O1 distance is longer and more distorted than the remaining H(D) bonds – the D2⋯O1 bond length measures 2.405 (5) Å, with an angle of 150.4 (4)°. This work shows that the D1⋯O1 and D6⋯O1 hydrogen bonds are slightly longer than the remaining two bonds, agreeing with the work of Adams & Small (1973 ▸).

#### 4.2.2. Thermal expansion of α-ammonium carbamate

The thermal expansion was determined for deuterated α-ammonium carbamate from the unit-cell parameters collected on HRPD at 4.2 K, in 5 K increments from 10 to 150 K, and in 10 K increments from 150 to 180 K.

**Figure 7:**
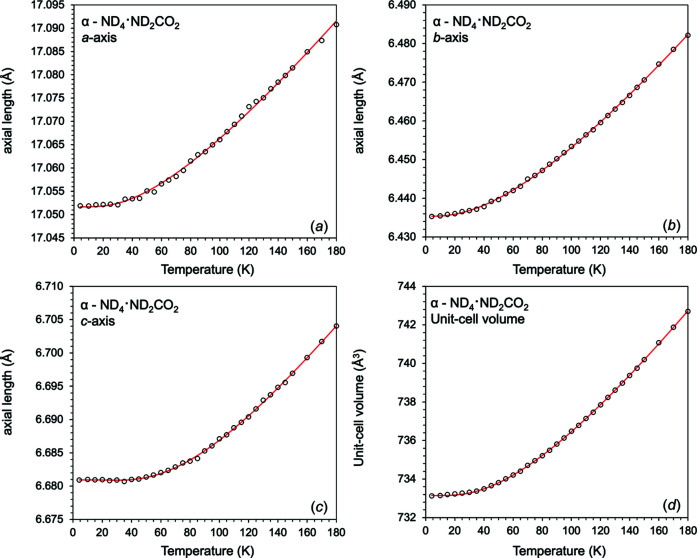
Unit-cell parameters (*a*)–(*c*) and unit-cell volume (*d*) of deuterated α-ammonium carbamate from 4.2 to 180 K. The solid symbols are from Le Bail refinements, with the solid red lines indicating the exponential model [equations (1) and (2)] fitted to the unit-cell parameters. A second-order (black line) Grüneisen–Debye model with a single Debye temperature [equation (S2)] is also fitted to the unit-cell volume. The second-order Grüneisen–Debye model is not distinguishable from the exponential model at this scale.

**Figure 8:**
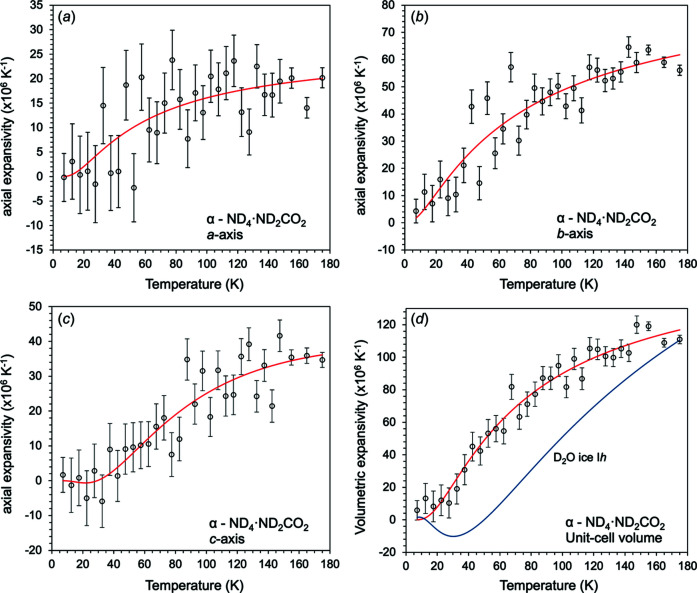
(*a*)–(*c*) Linear and (*d*) volume thermal expansion coefficients of deuterated α-ammonium carbamate with D_2_O ice I*h*. The white circles show values from the numerical derivative of the refined unit-cell data, the solid red lines indicate the expansivities computed from the exponential model [equation (1)] and the solid blue line is from a power-law/exponential model (see Fortes, 2018 ▸) fitted to the D_2_O ice I*h* present as a contaminant in the sample.

In order to model the thermal expansion to obtain useful thermoelastic parameters, a second-order Grüneisen–Debye model, with a single Debye temperature [equation (S2) in the supporting information], was used in least-squares fitting to the unit-cell volume, with the values *V*_0_, θ_D_, *Q* and *b* freely refined (further information regarding Grüneisen–Debye models can be found in the supporting information). The results of this fit are given in Table 2 ▸, and shown in Fig. 7 ▸. The calculated thermal expansion coefficients are shown in Fig. 8 ▸.

The thermal expansion appears to be dominated by low-frequency vibrational modes with characteristic temperatures in the range 200–230 K; this suggests that the phonon density of states has a cut-off at ∼17.2–19.8 meV (equivalent to wavenumbers ∼140–160 cm^−1^ or ∼4.2–4.8 THz, respectively). The fit of the Grüneisen–Debye model with a single Debye temperature also suggests that the heat capacity of α-ammonium carbamate does not approach the classical Dulong and Petit high-temperature limit (3*nk*_B_), but instead tends towards a high-temperature limit of ∼2*nk*_B_.

The value of *K*_0_/γ found from this Grüneisen–Debye fit to the unit-cell volume is 49 (1) GPa. This value is much greater than that calculated from fitting an equation of state to the outputs of the DFT calculations (see §4.2.4). The reason for this is most likely that a Debye model with a single Debye temperature does not properly describe the inter­nal energy of a mol­ecular solid such as α-ammonium carbamate (see supporting information). An alternative explanation is that γ ≠ 1; if a value of *K*_0_ is assumed from the high-pressure DFT simulations, where *K*_0_ = 16.5 GPa, equation (S2) gives γ ≈ 0.33 (1). It could also be that α-ammonium carbamate shows a temperature dependence of γ, which is assumed to be constant in this approximation.

Although Grüneisen–Debye models can provide useful thermoelastic parameters, they can be difficult to evaluate (due to their complexity and, in this case, lack of heat capacity data) and are only dimensionally correct for the unit-cell volume. Therefore, the thermal expansion data were also determined by fitting the unit-cell parameters using a linear combination of a power law and an exponential function:

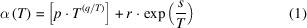

where *p* and *r* are scaling terms, and *q* and *s* are a ‘characteristic temperature’ indicative of the saturation behaviour of the function. The temperature dependence of the unit-cell parameters, or unit-cell volume, is then given by:

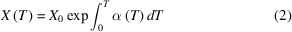

where *X*(*T*) is either an axial length or the unit-cell volume. The results (Table 3 ▸) have small uncertainties; furthermore, they lack the oscillatory behaviour of polynomial functions and give the correct behaviour at temperatures close to 0 K. As can be seen in Fig. 7 ▸, despite its *ad hoc* nature, the exponential model does an excellent job of fitting the experimental results, showing only extremely small differences from the Grüneisen–Debye model (nevertheless, we present the fitting of different Debye models in the supporting information).

The linear expansivities and the volumetric expansivity, derived from equation (1), are shown in Fig. 8 ▸. The symbols are obtained as point-by-point derivatives of the experimental unit-cell parameters, providing a useful visual indicator of the uncertainties due to their scatter.

There is a large degree of anisotropy in the structure upon warming; the greatest expansion is along the *b* axis, with the *a* and *c* axes having similar expansivities. The values of the volumetric thermal expansion coefficient are comparable with water ice and ammonium carbonate monohydrate at the same temperature (Fortes, 2018 ▸; Fortes *et al.*, 2014 ▸). We find no evidence for negative thermal expansion in the structure.

For making a simple density calculation as a function of temperature for planetary modelling, the density can be described using only the exponential function of equation (1) (as was sufficient to describe the volumetric expansion) and using:

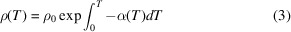

with the same values of *r* and *s* as determined from the fit to the unit-cell volume, and ρ_0_ = 1523.87 (2) kg m^−3^.

#### 4.2.3. DFT simulations of the structure of α-ammonium carbamate

The unit-cell volume at ambient pressure and 4.2 K, as measured on HRPD, is 733.126 (9) Å^3^. The zero-pressure athermal relaxation of α-ammonium carbamate from *CASTEP* yielded a unit-cell volume of 743.772 Å^3^, which equates to an approximately 1.5% overestimation of the measured value (Table 4 ▸). Although the simulated α-ammonium carbamate is protiated, the differences from the experimental structure, which is deuterated, are likely to be minor.

This difference in unit-cell volume can largely be attributed to the angle between the two herringbone-motif carbamate ions – the simulated value has this close to 90°, whereas the refined structure at 4.2 K has an angle greater than 90°. This means that the *b* axis has become longer and the *c* axis shorter in the simulated unit-cell parameters, resulting in the over- and underestimation of the individual unit-cell parameters. The result of relaxing α-ammonium carbamate made no significant changes to the structure. The bond lengths in the structure are only marginally different between the simulated and measured structures, *i.e.* the length of the carbamate ion is very similar, and the distance between centrosymmetric pairs of ions is 4.254 Å for the simulated and 4.304 (5) Å for the measured structures.

One thing to note is the distortion index of the ammonium tetra­hedra in the HRPD sample measured at 4.2 K, which is much larger than the other values in Table 1 ▸, but remains an order of magnitude smaller than that of Adams & Small (1973 ▸). Apart from the distortion of the tetra­hedra, the work on HRPD and the athermal zero-pressure DFT relaxation shows a very good agreement with the bonding geometry, such as the H(D)1⋯O1 and H(D)6⋯O1 hydrogen bonds being slightly longer than the remaining three bonds [excluding H(D)2⋯O1], and also agrees well with the work of Adams & Small (1973 ▸).

#### 4.2.4. Simulations of the high-pressure behaviour of α-am­monium carbamate

To provide information relevant to simulating α-ammonium carbamate in models of icy planetary inter­iors, the high-pressure behaviour was calculated by a series of relaxations over a pressure range between −0.9981 and +14.9952 GPa.

**Figure 9:**
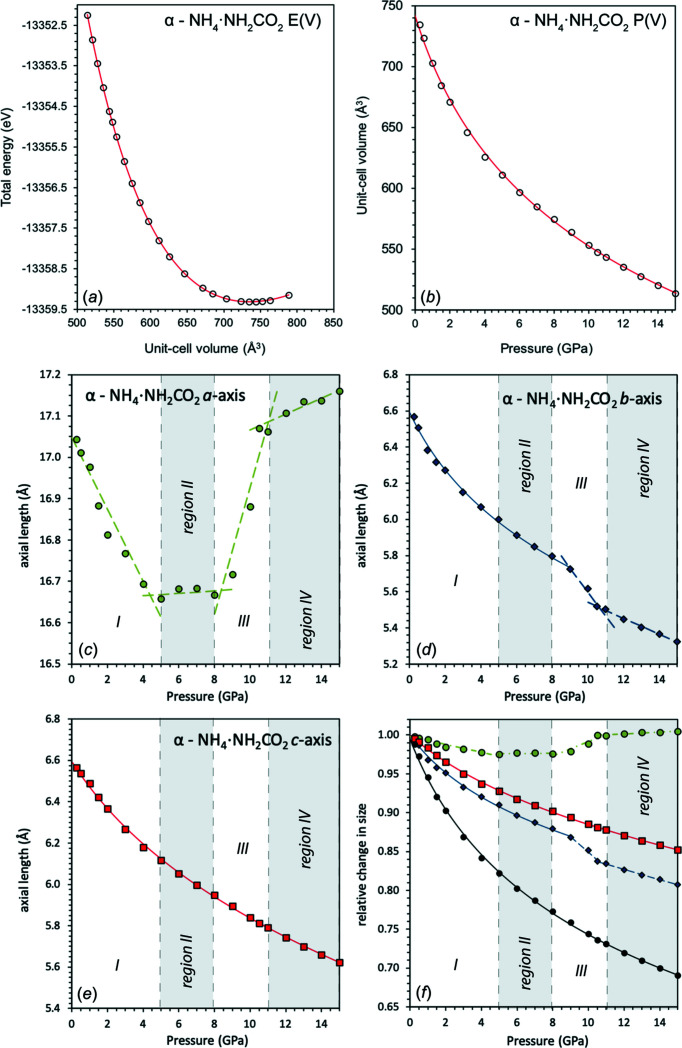
Plots of (*a*) energy–volume [*E*(*V*)] and (*b*) pressure–volume [*P*(*V*)] curves of α-ammonium carbamate, calculated using *CASTEP*. The solid lines show the best fit third-order Birch–Murnaghan equation of state [equations (S5) and (S6), BMEOS3]. (*c*)–(*f*) The relative change of the length of the unit-cell dimensions as a function of pressure of α-ammonium carbamate, showing the *a* axis (green), *b* axis (blue), *c* axis (red) and unit-cell volume (black). The shaded regions show the different compressive regimes explained in the text that are not obvious in the *E*(*V*) and *P*(*V*) curves. The dashed lines are simple linear fits to the points to guide the eye, where an equation of state cannot be fitted.

To qu­antify elastic parameters, an equation of state was fitted to the total energy against unit-cell volume [*E*(*V*)] and pressure against unit-cell volume [*P*(*V*)] curves (BMEOS3; Birch, 1952 ▸). These plots are given in Fig. 9 ▸, with the parameters from fitting to the calculated unit-cell parameters of α-ammonium carbamate given in Table 5 ▸. The values of *X*_0_, *K*_0_, *K* 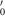 and *E*_0_ were allowed to freely refine. A parametric form of the Birch–Murnaghan equation of state was used to fit the compressibility along each of the unit-cell axes (for *b* ^3^ and *c* ^3^; the *a* axis is not fitted, which is explained next).

For making a simple density calculation as a function of pressure, the density was fitted using the equation of state used to fit *P*(*V*) points, where *X* = ρ/ρ_0_ (see supporting information). The fitted parameters are: ρ_0_ = 1394.2 kg m^−3^ (value fixed), *K*_0_ = 15.4 (3) GPa and *K* 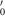 = 5.2 (1).

There is a high degree of anisotropy in the structure when compressed and, although not immediately apparent from examination of the *E*(*V*) and *P*(*V*) curves, there are some inter­esting and complex features in how the structure deforms in response to an external pressure, in particular along the *a* axis.

**Figure 10:**
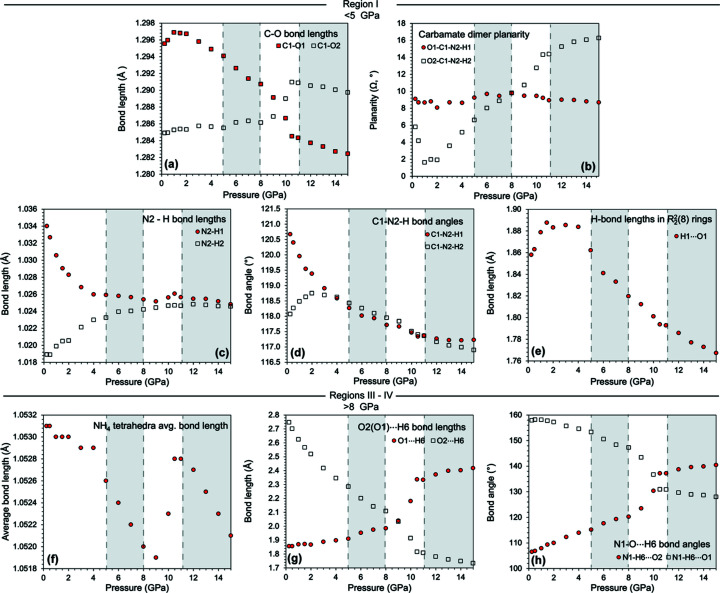
Structural features of α-ammonium carbamate as a function of pressure. Each of the panels shows different regions (shaded regions are II and IV, corresponding to 5–8 and >11 GPa, respectively) of the compressive behaviour that manifests along the *a* and *b* axes. Panels (*a*)/(*b*) show the behaviour of the carbamate mol­ecule changing up to pressures of 2 GPa, (*c*)–(*e*) show the changes up to 5 GPa and (*f*)–(*h*) show the changing bond geometry as the O2⋯H6 hydrogen bond is replaced by the O1⋯H6 hydrogen bond.

The initial loading shows subtle changes to the carbamate ions, manifesting in changes mostly along the *a* axis up to pressures of 5 GPa. During the initial loading, the carbamate ions reconfigure, particularly one of the H atoms on the N2 amino group. The N2—H1 and N2—H2 bonds shorten and lengthen, respectively, but in a rather smooth fashion, with the angles C1—N2—H1 and C1—N2—H2 also changing smoothly. This results in the carbamate ions themselves becoming more planar up to a pressure of 2 GPa before transitioning to a decrease in planarity (Fig. 10 ▸). The C1—O1 bond lengthens slightly, before shortening again. After this initial reconfiguration, the changes are mainly due to the H⋯O hydrogen bonds in the rings changing their behaviour, initially lengthening before shortening. Inter­estingly, these changes only manifest themselves in the lengths of the *a* and *b* axes, with barely any effect on the *c* axis.

This compressive behaviour continues up to a pressure of 5 GPa. However, along the *a* axis, between 5 and 8 GPa (region II), there is a small degree of negative linear com­pressibility (NLC), followed by an inversion to normal positive linear compressibility (PLC), averaging over this pressure range a small degree contraction along the *a* axis. The cause of this behaviour is a small rotation of the NH_4_^+^ tetra­hedra to accommodate the closing of the carbamate rings, causing the carbamate ions to move outwards along the *a* axis.

The largest NLC region in the *a* axis, however, occurs over the range 8–11 GPa (region III), continuing to a lesser extent up to ∼15 GPa (region IV), but this does not seem to translate into a significant change in the unit-cell volume, as can be observed in Fig. 10 ▸. The NLC apparent in the *a* axis manifests itself in the *b* and *c* axes in different ways: between 9.0 and 10.5 GPa, there is a marked change in slope of the *b* axis, which occurs at the same point as the end of the major part of the NLC; however, along the *c* axis, there is no obvious change in slope.

The cause of this large NLC is once again from the rotation of the NH_4_^+^ tetra­hedra, forcing the carbamate ions to move apart along the *a* axis. Alternating chains of ammonium tetra­hedra and carbamate ions along *b* likely reach a critical point, in that they cannot move closer together along either the *a* or the *b* axis. The only way to accommodate the increasing stress is therefore to rotate the ammonium tetra­hedra. As the carbamate ions had rotated at a lower pressure and had been packed closer together by the contraction along *b*, one of the O atoms was moved closer to the ammonium tetra­hedra, leading to the formation of a weak hydrogen bond. This is evident from the transfer of the H⋯O hydrogen bonds from the ammonium tetra­hedra to the carbamate ion (Fig. 10 ▸). As the NLC begins, the N1—H6⋯O1 hydrogen bond becomes highly strained because of the ammonium tetra­hedra rotating, and instead the bond changes to the O2 atom of the carbamate mol­ecule. This also causes the change in slope of the *b* axis.

The final transition at a critical pressure of 11.25 GPa (regions III and IV) is caused by the system reconfiguring to the rotated ammonium tetra­hedra, with the carbamate ions forced to translate along the *a* axis.

### 4.3. β-Ammonium carbamate

#### 4.3.1. DFT structure of β-ammonium carbamate

The zero-pressure athermal geometry optimization of the β-phase from *CASTEP* gave a unit-cell volume of 711.6 (2) Å^3^; although this equates to an approximately 3% overestimation of the measured value at 298 K (Kuhn *et al.*, 2007 ▸) (Table 6 ▸), the overestimation of the unit-cell volume is likely much larger, since, intuitively, it is expected the structure will contract upon cooling. An experiment at low temperature is needed to confirm the degree of overestimation of the unit-cell volume. However, since no experimental data are currently available, we present comparisons between the athermal simulation and the room-temperature experiment, where the differences can largely be attributed to the bond lengths; DFT calculated bond lengths for β-ammonium carbamate are compared to the experimental values in Table 7 ▸.

The N—H bond lengths given in Kuhn *et al.* (2007 ▸) are typically short due to the use of X-ray diffraction. The results of the present work, therefore, improve on the accuracy on the H-atom coordinates and are thus a much better determination of the hydrogen-bond lengths in the structure. Once again, this allows for more definitive statements regarding the structure and bonding to be made.

The previously published structure of β-ammonium carbamate indicated that it does not form the centrosymmetric dimer ring motifs 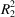 (8) seen in α-ammonium carbamate; instead, it forms one-dimensional chains along the mirror plane by the hydrogen bonding of a nitrile H atom to one of the carbonyl O atoms (H4⋯O2) at *c*/2 inter­vals. The remaining nitrile H atom (H3), in this instance, is unlikely to form a hydrogen bond, due to the N2—H3⋯O2 hydrogen bond having a small angle (115°) and long length (∼3 Å). Each carbonyl O atom has two out-of-plane hydrogen bonds to two separate ammonium tetra­hedra, which lie either side of the mirror plane, with the tetra­hedra midway between the chains.

**Figure 11:**
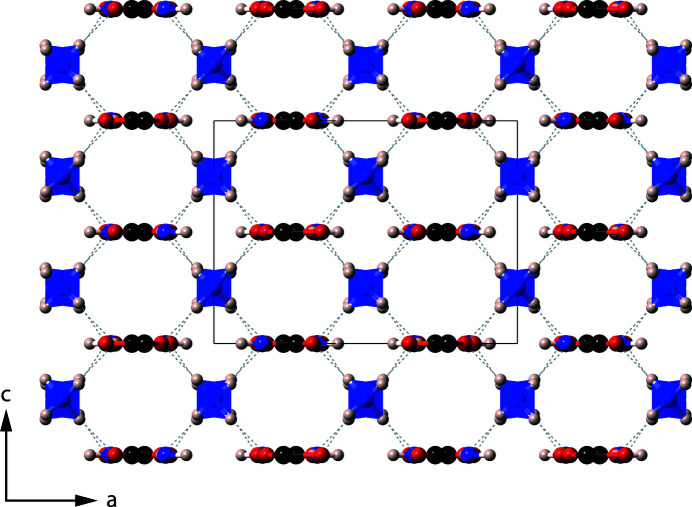
View of the DFT-simulated structure of β-ammonium carbamate parallel to the *b* axis. The grey dashed lines are H⋯O hydrogen bonds and the solid black line is the unit cell.

**Figure 12:**
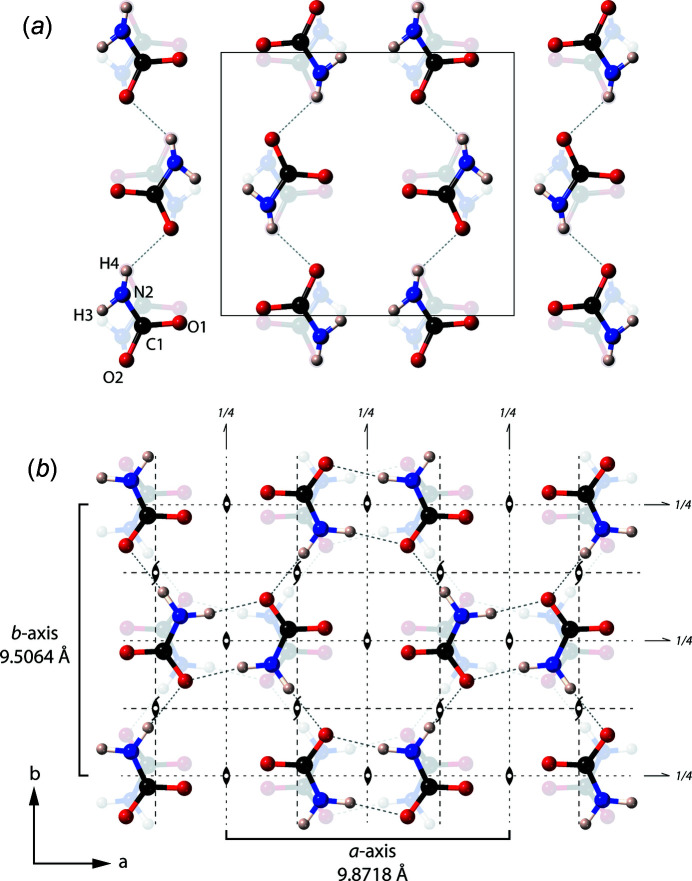
Comparison of the hydrogen-bond motif of β-ammonium carbamate in the *ab* plane from (*a*) an X-ray diffraction experiment (Kuhn *et al.*, 2007 ▸) and (*b*) the zero-pressure structure from *CASTEP* with symmetry operators overlain. The relaxed structure shows the same hexa­gonal-ring motif present in the α-phase.

However, in the DFT-relaxed structure (Fig. 11 ▸), the carbamate mol­ecules have rotated, resulting in the H atoms changing position. The N2—H3⋯O2 hydrogen bond has become quite linear, with an angle of ∼172°, and is much shorter, at 2.074 Å. The relaxing of the H atoms has altered the bonding geometry so that, now, β-ammonium carbamate forms the same hexa­gonal ring motif, a double two-centre hydrogen-bonded 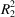 (8) ring that is seen in the α-phase, albeit with a different motif layout to the latter (see Fig. 12 ▸); the motifs instead lay on mirror planes, but are offset by the two glide planes in the structure. These planar 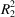 (8) rings are joined *via* ammonium ions at *z* = 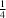 and *z* = 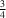 .

It can also be seen in the experimental values that the ammonium tetra­hedra are extremely distorted (Table 8 ▸). In the relaxed structure, the NH_4_ tetra­hedra have a smaller distortion, with each of the H atoms donating a hydrogen bond to different carbamate O atoms. The length of the H1⋯O1 and H2⋯O2 hydrogen bonds, which form the out-of-plane bonds between the NH_4_ tetra­hedra and planar NH_2_CO_2_ ions, are shorter than the remaining two hydrogen bonds that form parallel to the planes.

#### 4.3.2. Simulations of the high-pressure behaviour of β-ammonium carbamate

As with α-ammonium carbamate, the high-pressure behaviour of β-ammonium carbamate was simu­lated to provide information relevant for planetary models. A series of static relaxations were performed over a pressure range between −1.9977 and +10.025 GPa.

**Figure 13:**
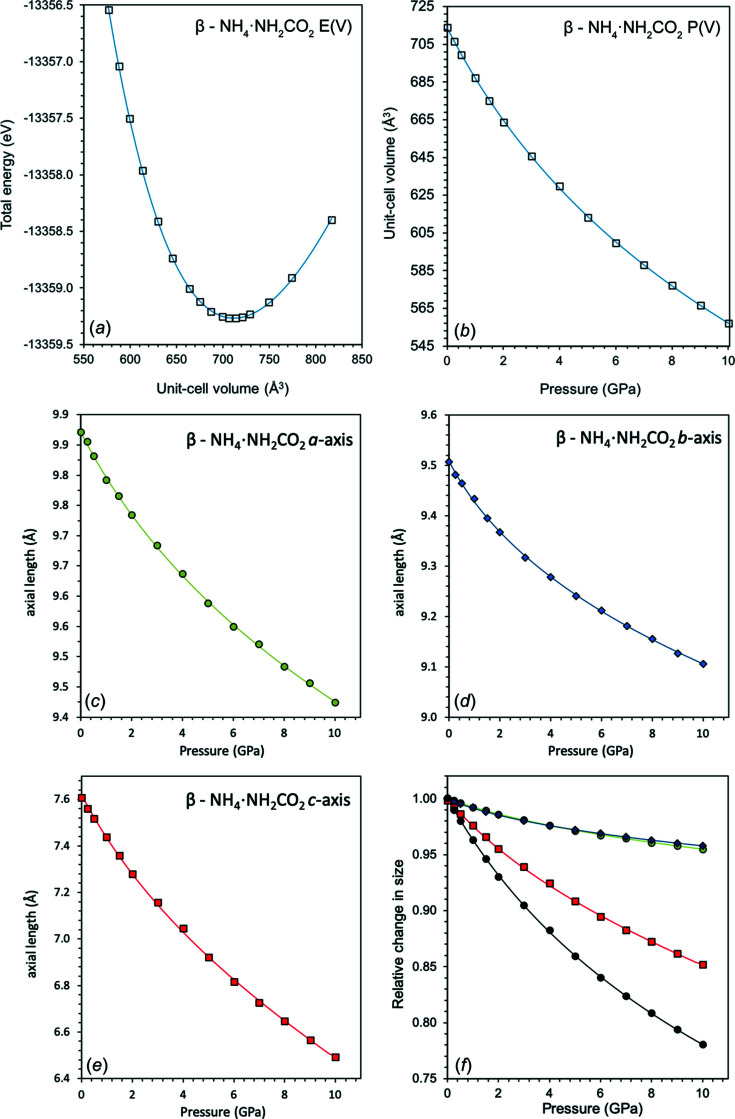
Plots of (*a*) energy–volume [*E*(*V*)] and (*b*) pressure–volume [*P*(*V*)] curves of β-ammonium carbamate, calculated using *CASTEP*. The solid lines show the best-fit third-order Birch–Murnaghan equation of state (BMEOS3) [equations (S5) and (S6)]. (*c*)–(*f*) The unit-cell dimensions of β-ammonium carbamate as a function of pressure. The solid lines are fitted BMEOS3 [equation (S5)], corresponding to the *a* axis (green), *b* axis (blue), *c* axis (red) and unit-cell volume (black).

To gain information on the bulk elastic parameters, *E*(*V*) and *P*(*V*) curves were fitted with a BMEOS3 equation of state, with the plots given in Fig. 13 ▸ and values of the fit in Table 8 ▸. The values of *X*_0_, *K*_0_, *K* 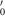 and *E*_0_ were allowed to freely refine. Plots of the unit-cell parameters as a function of pressure are given in Fig. 13 ▸. A parametric form of the Birch–Murnaghan equation of state was used to fit the compressibility along each of the unit-cell axes (for *a* ^3^, *b* ^3^ and *c* ^3^). The density as a function of pressure was fitted using the same equation of state used to fit *P*(*V*) points. The fitted parameters are: ρ_0_ = 1452.7 (8) kg m^−3^, *K*_0_ = 24.2 (3) GPa and *K* 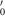 = 4.11 (8).

In β-ammonium carbamate, the *a* and *b* axes compress at a much slower rate than the *c* axis (Fig. 13 ▸). The compression seen along the *a* and *b* axes is caused by the similar hydrogen-bond geometry in both directions from the 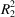 (8) ring motifs of the carbamate ions. The structure is much less stiff perpendicular to the planar layers, in which the planar layers of carbamate ions are linked by ammonium tetra­hedra halfway between. This can be seen in the compression of the medium-range hydrogen bonds that connect the layers of carbamate ions *via* ammonium tetra­hedra; the O1⋯H1 and O2⋯H2 hydrogen bonds compress by 5.32 and 6.65%, respectively, over a pressure range of 0 to 10.025 GPa.

### 4.4. Thermodynamics

Since the total inter­nal energies of both structures have been simulated using DFT as a function of pressure, it is possible to assess the relative stability of both phases in order to determine where a phase change may occur as a function of pressure.

**Figure 14:**
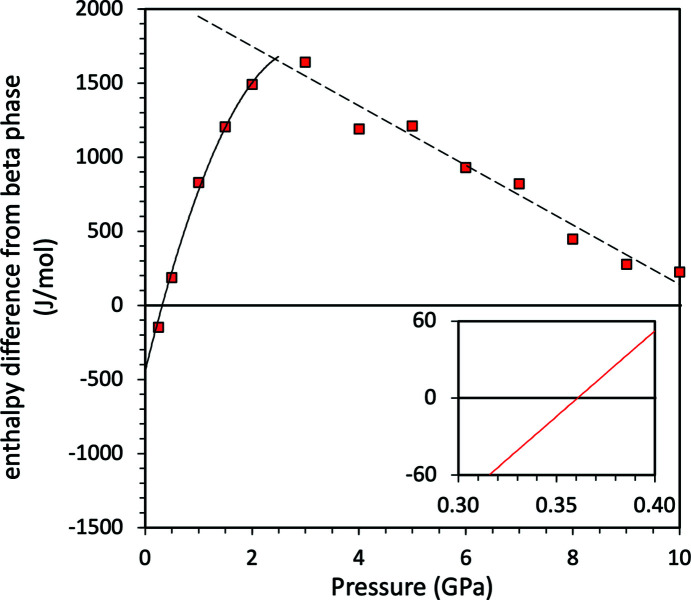
Calculated enthalpy difference between β-ammonium carbamate (normalized to zero) and α-ammonium carbamate (red squares). Enthalpies were output from the dispersion-corrected simulations of *CASTEP*, with the lines a simple polynomial fit (solid) and a linear fit (dashed) to the points.

The relative stability of the two phases is assessed by calculating the enthalpy (*H*) as a function of pressure for each phase, calculated using *H* = *U* + *PV*; since the simulations are athermal the enthalpy is equal to the Gibbs free energy. The enthalpies are compared in Fig. 14 ▸. The α-polymorph has the lowest enthalpy of the two phases and is hence the stable phase at zero-pressure in the athermal limit, with a difference of 399.33 J mol^−1^ with respect to the β-polymorph. When increasing the pressure, the enthalpies of the two phases cross; the transition pressure between the two phases, where Δ*H* = 0, is ∼0.4 GPa. The β-polymorph has the lowest enthalpy above ∼0.4 GPa and is then the stable phase in the athermal limit. This trend, of the α-polymorph having an increasing enthalpy difference relative to the β-polymorph, continues up to pressures of ∼2 GPa. However, as the α-phase starts to undergo subtle structural changes above this pressure, the trend reverses and the enthalpy difference between the two phases begins to decrease.

## 5. Discussion

### 5.1. Thermoelastic behaviour

The presence of negative linear compression in α-ammonium carbamate is rather uncommon, since most materials compress in all directions when hydro­statically stressed. It seems rather curious that the negative linear compression behaviour calculated to exist in the α-phase is not seen in β-ammonium carbamate, since both phases consist of the same centrosymmetric 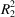 (8) ring motifs. If it were found in the β-structure, a ‘wine-rack’ motif that forms between the carbamate ions on the mirror planes and the ammonium ions that form halfway between these layers would cause the carbamate ions on the mirror planes to squeeze closer together, with the ammonium ions moving further apart to accommodate this change. Further experimental work, in particular a high-pressure study, is needed to verify the existence of the calculated NLC seen in α-ammonium carbamate, whether a phase transition would occur before the NLC region and to investigate the phase relationship between the two known phases.

The measured thermal expansion correlates well with the calculated high-pressure behaviour of α-ammonium carbamate. The high degree of anisotropy under pressure in both these polymorphs is due to details in the structure: α-ammonium carbamate’s *b* axis is the most compressible, due to few hydrogen bonds forming between layers of ammonium tetra­hedra and carbamate ions, which run parallel to the *c* axis, and hence provide little resistance to compression; β-ammonium carbamate is most compressible along *c* due to planar layers of carbamate ions running in chains in the *ab* plane, with ammonium tetra­hedra forming the inter­layer halfway between these parallel chains. The *a* and *b* axes have lower but similar compressibility due these to planar layers of carbamate ions.

**Figure 15:**
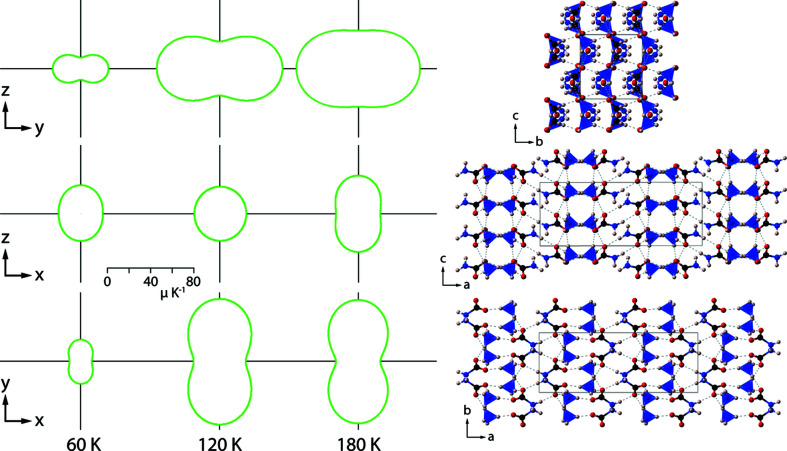
Projections of the thermal expansion coefficient representation surface in the *yz* (top), *xz* (middle) and *xy* (bottom) planes at 60, 120 and 180 K. Solid green lines are positive values. Corresponding projections of the structure of α-ammonium carbamate are shown on the right.

In terms of thermal expansion, the direction of greatest thermal expansion is perpendicular to the corrugated sheets of alternating ammonium tetra­hedra and centrosymmetric car­bam­ate ionic dimers, whereas the minimum thermal expansion is in the plane of these alternating sheets. The inter­mediate value is parallel to stacks of centrosymmetric carbamate ions running along *c*, connected by chains of ammonium tetra­hedra. The limited bonding between the corrugated sheets running along the *b* axis provides little resistance to expansion, with a three-dimensional bonding network running in the planes of these sheets resisting expansion (Fig. 15 ▸). Based upon the DFT calculations, we would expect that β-ammonium carbamate should have a smaller thermal expansivity than α-ammonium carbamate, due to its lower compressibility.

When the two phases of ammonium carbamate are compared against thermal expansion measurements of other compounds in this ternary system, the high degree of anisotropy is comparable. The ammonium carbonates are highly anisotropic, typically with one unique axis, and two axes that behave similarly, due to planar layers of inter­connected ammonium tetra­hedra. Indeed, in ammonium carbonate monohydrate, the *c* axis has been shown from variable-temperature neutron single-crystal data to exhibit negative linear expansion, whilst the *a* and *b* axes exhibit large positive expansivities of similar magnitude (Fortes *et al.*, 2014 ▸).

### 5.2. Planetary modelling

The work presented here provides some important values for use in planetary modelling: the density as a function of pressure has been calculated for both structures of ammonium carbamate, accurate crystal structures of both phases of am­monium carbamate for further *ab initio* simulations have been obtained, and the thermal expansion coefficients of α-ammonium carbamate are now also known. However, further work is still required to understand the properties of ammonium carbamate in planetary environments. In particular, vibrational spectra are required for unambiguous identification on planetary surfaces, as is a study of the *P*–*T* phase diagram under conditions applicable to icy bodies, *i.e.* high pressure and low temperature.

## 6. Conclusion

We report dispersion-corrected density functional theory simulations of both α- and β-ammonium carbamate, the first neutron powder diffraction study of α-ammonium carbamate, together with the first structure refinement at non-ambient tem­perature, measured at 4.2 K. We also carried out the first thermal expansion measurements of α-ammonium carbamate (from 4.2 to 180 K) and have shown how the magnitudes of the axial expansivities are related to the crystal structure. We find evidence for the unusual property of NLC in the *a* axis of α-ammonium carbamate above 5 GPa, with the largest degree of negative expansivity occurring between 9 and 10.5 GPa. This work has provided important parameters to be incorporated into structural models of icy bodies in the Solar System. Further experiments at high pressure are needed to investigate the calculated negative linear compressibility in α-am­monium carbamate and the possible phase transition between the two known polymorphs.

## Supplementary Material

Crystal structure: contains datablock(s) global, ammonium_carbamate_publ, ammonium_carbamate, water_ice, 59833_backscattering_banks. DOI: 10.1107/S2052520622002645/xk5092sup1.cif

Additional tables and information. DOI: 10.1107/S2052520622002645/xk5092sup2.pdf

CCDC references: 2162678, 2162679

## Figures and Tables

**Table 1:** Comparison of bond lengths, polyhedral volumes, various distortion metrics and hydrogen-bond parameters (Å, °) in α-ammonium carbamate The values are from the outputs of *CASTEP* and measurements on HRPD, a powder X-ray diffraction experiment (Baisch *et al.*, 2006 ▸) and a single-crystal X-ray diffraction experiment (Adams & Small, 1973 ▸). Note that the HRPD sample was deuterated. Atomic labels in Baisch *et al.* (2006 ▸) correspond to: H11 = H3, H12 = H2, H22 = H5, H23 = H6, H24 = H4 and N2 = N1, according to the labelling of Adams & Small (1973 ▸). The distortion index is based on bond lengths in the polyhedra and was defined by Baur (1974 ▸). ‘Quadratic elongation’ gives a quantative measure of polyhedral distortion, which is independent of the effective polyhedron size (Robinson *et al.*, 1971 ▸), and ‘bond-angle variance’ gives a quantative measure of how the bond angles in the tetra­hedra deviate from the ideal value of 109.28 – lower values equate to bond angles closer to the ideal (Robinson *et al.*, 1971 ▸).

	DFT athermal	HRPD neutron 4.2 K	X-ray 293 (2) K (Baisch *et al.*, 2006 ▸)	X-ray 295 K (Adams & Small, 1973 ▸)
C1—O1	1.300	1.247 (5)	1.269 (3)	1.270 (5)
C1—O2	1.285	1.272 (5)	1.266 (2)	1.279 (4)
C1—N2	1.361	1.362 (4)	1.340 (3)	1.348 (5)
N2—H(D)1	1.034	1.018 (5)	0.84 (3)	0.85 (6)
N2—H(D)2	1.019	1.002 (5)	0.81 (3)	0.83 (7)
N1—H(D)3	1.044	1.067 (4)	0.94 (4)	1.00 (5)
N1—H(D)4	1.055	1.052 (5)	0.93 (3)	0.85 (6)
N1—H(D)5	1.051	1.076 (5)	0.93 (3)	0.91 (6)
N1—H(D)6	1.064	1.049 (5)	0.93 (3)	0.75 (6)
NH(D)_4_ volume	0.5987	0.6106	0.4166	0.3205
Distortion index	0.0057	0.0098	0.0028	0.08704
Quadratic elongation	1.001	1.0026	1.0026	1.0615
Bond-angle variance	3.4483	10.2483	9.7185	114.3099
				
H(D)1⋯O1	1.863	1.949 (5)	2.16 (3)	2.12 (5)
H(D)2⋯O1†	2.516	2.405 (5)	2.70 (3)	2.64 (6)
H(D)3⋯O2	1.674	1.690 (5)	1.82 (3)	1.77 (5)
H(D)4⋯O2	1.762	1.792 (5)	1.93 (3)	2.13 (5)
H(D)5⋯O1	1.767	1.779 (5)	1.93 (3)	1.93 (6)
H(D)6⋯O1	1.862	1.914 (6)	2.03 (3)	2.19 (6)
N2—H(D)1⋯O1	177.1	171.2 (4)	174.7 (8)	174 (5)
N2—H(D)2⋯O1†	148.0	150.4 (4)	149.9 (8)	158 (1)
N1—H(D)3⋯O2	170.2	170.1 (4)	171.4 (8)	166 (4)
N1—H(D)4⋯O2	164.3	166.2 (4)	162.4 (8)	138 (5)
N1—H(D)5⋯O1	170.3	170.5 (4)	168.5 (8)	173 (4)
N1—H(D)6⋯O1	157.5	158.4 (4)	155.4 (7)	158 (5)

Notes: (†) in Adams & Small (1973 ▸), this is suggested to not be a hydrogen bond.

**Table 2:** Parameters obtained by fitting a second-order Grüneisen–Debye model with a single Debye temperature [equation (S2)] to the unit-cell volume of α-ammonium carbamate between 4.2 and 180 K

	*V*_0_ (Å)	*K*_0_/γ (GPa)	θ_D_ (K)	*Q* (J)	*b*	Adjusted *R* ^2^
Second-order	733.15 (1)	49 (1)	208 (5)	3.60 (9) ×10^−17^	10 (1)	0.99991

**Table 3:** Parameters obtained by fitting of equations (1) and (2) to the unit-cell parameters and unit-cell volume of α-ammonium carbamate between 4.2 and 180 K

	*X*_0_ (Å, Å^3^)	*P* (×10^−4^)	*q*	*r* (×10^−4^)	*s*	Adjusted *R* ^2^
*a* axis	17.0517 (2)	–	–	0.268 (8)	−51 (2)	0.99827
*b* axis	6.43539 (9)	0.95 (1)	−14.5 (3)	–	–	0.99973
*c* axis	6.68077 (6)	–	–	0.74 (2)	−113 (3)	0.99903
Volume (Å^3^)	733.148 (9)	–	–	1.65 (1)	−60.9 (6)	0.99993

**Table 4:** A comparison of the unit-cell parameters of α-ammonium carbamate from the DFT athermal zero-pressure relaxation using *CASTEP*, from an X-ray single-crystal diffraction (SXD) experiment at room temperature (Adams & Small, 1973 ▸) and from a powder X-ray diffraction experiment (PXRD) at room temperature (Baisch *et al.*, 2006 ▸)

Formula	(NH_4_)^+^·(NH_2_CO_2_)^−^				
Crystal system	Ortho­rhom­bic				
Space group	*Pbca*				
*Z*	8				
Reference	This work	This work	This work	Adams & Small (1973 ▸)	Baisch *et al.* (2006 ▸)
Method	DFT	Neutron	Neutron	X-ray	X-ray
*T* (K)	Athermal	4.2	180	295	293 (2)
*a* axis (Å)	17.0796	17.0519 (2)	17.0908 (2)	17.121 (6)	17.119 (4)
*b* axis (Å)	6.61022	6.43518 (7)	6.48215 (8)	6.531 (2)	6.535 (2)
*c* axis (Å)	6.58788	6.68093 (8)	6.7040 (1)	6.742 (3)	6.754 (2)
Volume (Å^3^)	743.772	733.122 (9)	742.71 (1)	753.9 (5)	755.73 (3)

**Table 5:** Fitted third-order Birch–Murnaghan equation of state parameters [equations (S5) and (S6)] of α-ammonium carbamate Fits to equation (S5) were achieved using only those pressure values that were greater than 0 GPa. For the *b* and *c* axes, the values fitted were *b* ^3^ and *c* ^3^. No equation of state could be fitted for the *a* axis due to the presence of negative linear compression.

	*X*_0_ (Å, Å^3^)	*K*_0_ (GPa)	*K* 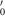	*K* 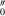 (GPa^−1^)	*E*_0_ (eV mol­ecule^−1^)
BMEOS3, *P*(*b*)†	6.594 (9)	10.3 (4)	3.6 (1)	−0.35 (2)*	
BMEOS3, *P*(*c*)	6.595 (7)	16.2 (4)	3.08 (5)	−0.236 (4)*	
BMEOS3, *P*(*V*)	744 (2)	16.5 (4)	5.0 (1)	−0.36 (3)*	
BMEOS3, *E*(*V*)	743.7 (6)	16.0 (3)	5.35 (9)	−0.44 (3)*	−1669.9149 (4)

Notes: (†) fitted to points below 9 GPa due to change of slope; (*) derived from fitted values of *K*_0_ and *K* 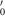 .

**Table 6:** Comparison of bond lengths (Å), polyhedral volumes, various distortion metrics and hydrogen-bond parameters (Å, °) in β-ammonium carbamate The β-ammonium carbamate values are from the outputs of *CASTEP* and a powder X-ray diffraction experiment

	DFT athermal	X-ray 298 K (Kuhn *et al.*, 2007 ▸)
C1—O1	1.300	1.287 (9)
C1—O2	1.285	1.320 (9)
C1—N2	1.361	1.294 (9)
N2—H1	1.034	0.89 (6)
N2—H2	1.019	0.83 (6)
N1—H1	1.044	0.99 (4)
N1—H2	1.055	1.12 (4)
N2—H3	1.051	0.89 (7)
N2—H4	1.064	0.83 (6)
NH_4_ volume	0.5987	0.5883
Distortion index	0.0057	0.0600
Quadratic elongation	1.001	1.0231
Bond-angle variance	3.4483	65.0215
		
H1⋯O1	1.710	1.87 (4)
H2⋯O2	1.804	1.77 (4)
H3⋯O2	2.074	3.01 (6)
H4⋯O2	2.091	2.15 (6)
N1—H1⋯O1	171.31	100.5 (6)
N1—H2⋯O2	165.40	157.2 (8)
N2—H3⋯O2	171.67	115.0 (6)
N2—H4⋯O2	176.67	143 (2)

**Table 7:** A comparison of the unit-cell parameters of β-ammonium carbamate from the DFT athermal zero-pressure relaxation using *CASTEP* and a powder X-ray diffraction (PXRD) experiment at room temperature (Kuhn *et al.*, 2007 ▸)

Formula	(NH_4_)^+^·(NH_2_CO_2_)^−^	
Crystal system	Ortho­rhom­bic	
Space group	*Ibam*	
*Z*	8	
Reference	This work	Kuhn *et al.* (2007 ▸)
Method	DFT	X-ray
*T* (K)	Athermal	298
*a* axis (Å)	9.8718	10.1428
*b* axis (Å)	9.5064	9.1579
*c* axis (Å)	7.6071	7.4485
Volume (Å^3^)	713.89	691.87

**Table 8:** Fitted equation of state parameters [equations (S5) and (S6)] of β-ammonium carbamate Fits to equation (S5) were achieved using only those pressure values that were greater than 0 GPa. For the axial values, the cubes of the axes were fitted.

	*X*_0_ (Å, Å^3^)	*K*_0_ (GPa)	*K* 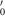	*K* 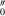 (GPa^−1^)	*E*_0_ (eV mol­ecule^−1^)
*P*(*a*)	9.872 (3)	39 (2)	9.3 (6)	−0.9 (2)	
*P*(*b*)	9.508 (3)	32 (1)	17 (1)	−6 (1)	
*P*(*c*)	7.620 (8)	12.2 (3)	2.76 (5)	−0.345 (4)	
*P*(*V*)	713.6 (5)	24.4 (4)	4.09 (9)	−0.164 (7)	
*E*(*V*)	711.6 (2)	23.3 (2)	4.51 (8)	−0.200 (7)	−1669.9084 (3)
