# Supplementary material for: Ab initio simulations of α- and β-ammonium carbamate (NH4·NH2CO2), and the thermal expansivity of deuterated α-ammonium carbamate from 4.2 to 180 K by neutron powder diffraction
Source: Acta Crystallogr B Struct Sci Cryst Eng Mater. 2022 Apr 30;78(Pt 3):459–75. doi: 10.1107/S2052520622002645 (PMC9254591; doi:10.1107/S2052520622002645)
Supplement: Supplementary file 2 [file b-78-00459-sup2.pdf]

STRUCTURAL SCIENCE
CRYSTAL ENGINEERING
MATERIALS

Volume 78 (2022)

Supporting information for article:

Ab initio simulations of α- and β-ammonium carbamate (NH4·NH2CO2), and the thermal expansivity of deuterated α-ammonium carbamate from 4.2 to 180 K by neutron powder diffraction

Christopher M. Howard, Ian G. Wood, Kevin S. Knight and A. Dominic Fortes

CONTENTS

S1 Deuterated α-ammonium carbamate lattice parameters as a function of T

Table S1: Deuterated α-ammonium carbamate lattice parameters as a function of T

S2 DFT-simulated lattice parameters of α- and β-ammonium carbamate

Table S2: DFT-simulated lattice parameters of α-ammonium carbamate

Table S3: DFT-simulated lattice parameters of β-ammonium carbamate

S3 Fitting Grüneisen-Debye models to the thermal expansion

Table S4: Comparison of Grüneisen-Debye models fitted to the unit-cell volume data as determined on HRPD

S4 Fitting equations of state to the DFT simulations of α-ammonium carbamate and β-ammonium carbamate

S1. Deuterated α-ammonium carbamate lattice parameters as a function of T

Table S1

Refined lattice parameters as a function of temperature from neutron powder diffraction data measured on HRPD.

T (K)	*a* (Å)	*b* (Å)	*c* (Å)	V (Å^3)
4.2	17.05189(15)	6.43531(7)	6.68093(7)	733.126(9)
10	17.05185(46)	6.43545(15)	6.68099(18)	733.147(24)
15	17.05211(47)	6.43581(15)	6.68095(19)	733.195(24)
20	17.05214(49)	6.43604(16)	6.68098(19)	733.225(25)
25	17.05224(47)	6.43655(15)	6.68081(18)	733.269(24)
30	17.05210(48)	6.43684(15)	6.68090(18)	733.307(24)
35	17.05334(46)	6.43718(15)	6.68071(18)	733.377(24)
40	17.05340(46)	6.43786(14)	6.68101(18)	733.490(24)
45	17.05349(43)	6.43923(14)	6.68105(17)	733.651(22)
50	17.05508(43)	6.43970(14)	6.68136(17)	733.810(23)
55	17.05489(41)	6.44118(13)	6.68168(16)	734.006(21)
60	17.05662(41)	6.44200(13)	6.68202(16)	734.211(21)
65	17.05743(38)	6.44311(12)	6.68237(15)	734.412(20)
70	17.05820(38)	6.44496(12)	6.68289(16)	734.712(20)
75	17.05948(36)	6.44593(12)	6.68349(15)	734.945(19)
80	17.06151(37)	6.44721(12)	6.68374(15)	735.206(20)
85	17.06285(37)	6.44881(11)	6.68414(14)	735.490(19)
90	17.06351(35)	6.45025(11)	6.68531(14)	735.811(18)
95	17.06497(34)	6.45180(11)	6.68604(14)	736.131(18)
100	17.06608(32)	6.45342(10)	6.68710(13)	736.480(17)
105	17.06783(32)	6.45480(11)	6.68771(13)	736.781(17)
110	17.06935(33)	6.45640(11)	6.68877(13)	737.146(17)
115	17.07115(32)	6.45773(11)	6.68958(14)	737.465(18)
120	17.07317(31)	6.45958(10)	6.69040(13)	737.854(17)
125	17.07429(29)	6.46139(10)	6.69160(12)	738.241(16)
130	17.07507(28)	6.46308(9)	6.69291(11)	738.613(15)
135	17.07699(26)	6.46479(9)	6.69372(11)	738.981(14)
140	17.07842(25)	6.46659(8)	6.69483(11)	739.370(14)
145	17.07984(29)	6.46868(9)	6.69554(11)	739.750(14)
150	17.08150(25)	6.47058(8)	6.69694(10)	740.194(14)
160	17.08494(25)	6.47469(8)	6.69931(11)	741.075(14)
170	17.08733(26)	6.47851(9)	6.70171(11)	741.883(14)
180	17.09078(24)	6.48215(8)	6.70403(10)	742.706(13)

S2. DFT-simulated lattice parameters of α- and β-ammonium carbamate

Table S2

Lattice parameters and total energy per unit-cell as a function of pressure of α-ammonium carbamate from DFT-simulations

P (GPa)	E (eV)	*a* (Å)	*b* (Å)	*c* (Å)	V (Å^3)
-0.998	-13359.15	17.238019	6.855010	6.671210	788.3155
-0.503	-13359.28	17.152659	6.715171	6.622883	762.8438
-0.252	-13359.31	17.122348	6.659597	6.598044	752.3613
-0.005	-13359.32	17.079629	6.610215	6.587884	743.7722
0.250	-13359.32	17.043552	6.566966	6.564531	734.7314
0.502	-13359.30	17.011360	6.506863	6.537128	723.5986
1.000	-13359.24	16.977022	6.382477	6.489191	703.1393
1.499	-13359.11	16.883396	6.315644	6.420637	684.6294
1.999	-13358.98	16.812261	6.271282	6.365537	671.1468
2.998	-13358.62	16.767465	6.150297	6.266224	646.2037
4.005	-13358.20	16.693124	6.068236	6.179217	625.9412
4.998	-13357.81	16.657856	5.999637	6.116599	611.2996
5.998	-13357.33	16.681941	5.911793	6.051427	596.7928
6.995	-13356.87	16.683289	5.849037	5.996513	585.1468
8.001	-13356.39	16.667095	5.796738	5.947697	574.6354
9.002	-13355.85	16.716496	5.724478	5.896080	564.2148
9.995	-13355.24	16.880489	5.616252	5.838950	553.5612
10.504	-13354.88	17.069944	5.520063	5.812119	547.6595
11.000	-13354.62	17.061681	5.503150	5.790365	543.6747
12.003	-13354.03	17.107452	5.449049	5.742510	535.3130
13.002	-13353.44	17.134710	5.403238	5.698278	527.5632
14.000	-13352.85	17.137182	5.366489	5.659914	520.5225
14.995	-13352.25	17.159925	5.325087	5.621503	513.6823

Table S3

Lattice parameters and total energy per unit-cell as a function of pressure of β-ammonium carbamate from DFT-simulations

P (GPa)	E (eV)	*a* (Å)	*b* (Å)	*c* (Å)	V (Å^3)
-1.998	-13358.3996	10.102794	9.973565	8.107420	816.9107
-1.499	-13358.9094	10.004730	9.724473	7.958345	774.2732
-0.995	-13359.1251	9.953700	9.633400	7.814665	749.3324
-0.504	-13359.2334	9.909009	9.555925	7.697727	728.8958
-0.247	-13359.2563	9.889303	9.525446	7.654358	721.0407
0.002	-13359.2665	9.871799	9.506395	7.607130	713.8928
0.249	-13359.2664	9.855471	9.481256	7.560547	706.4745
0.501	-13359.2549	9.832140	9.463939	7.516987	699.4615
0.993	-13359.2102	9.792418	9.432917	7.438969	687.1456
1.499	-13359.1238	9.765817	9.395165	7.358618	675.1640
2.001	-13359.0085	9.734164	9.367428	7.279055	663.7339
3.000	-13358.7403	9.684457	9.317083	7.156486	645.7361
4.000	-13358.4128	9.637117	9.277578	7.044674	629.8580
5.000	-13357.9606	9.588755	9.240414	6.921291	613.2545
6.000	-13357.5056	9.549818	9.211290	6.816477	599.6192
6.999	-13357.0419	9.520547	9.181399	6.726126	587.9437
7.999	-13356.5428	9.483973	9.155013	6.645956	577.0411
9.007	-13356.0046	9.456394	9.126776	6.565979	566.6860
10.003	-13355.4509	9.424122	9.105415	6.491856	557.0697

S3. Fitting Grüneisen-Debye models to the thermal expansion

Following Wallace (1998), the following expressions can be used to model the thermal expansion of a crystal in terms of its internal energy to first-order:

$$V(T) = V_0 + \frac{\gamma U(T)}{K_0} \qquad (Eq. S1)$$

where V_0 is the unit-cell volume at zero-temperature, and to second-order,

$$V(T) = V_0 + \frac{V_0 U(T)}{Q - bU(T)} \qquad (Eq. S2)$$

where $Q = V_0 K_0/\gamma$, $b = (K_0' - 1)/2$, K_0 and K_0' are the bulk modulus and its first derivative with respect to pressure at $T = 0$ respectively, and γ is a Grüneisen parameter.

There then comes a choice between the use of Einstein models and Debye models to define the internal energy, both of which have their own advantages. Although Einstein models are usually simpler to compute, they are less valid for low temperature measurements. Debye models are more complicated to fit but offer more useful parameters that could be applicable to planetary models; therefore, it was decided a Debye model was to be used.

The internal energy, $U(T)$, calculated using a Debye approximation is,

$$U(T) = 9Nk_B T \left(\frac{T}{\theta_D}\right)^3 \int_0^{\theta_D/T} \frac{x^3}{e^x - 1} dx \qquad (Eq. S3)$$

where N is the number of atoms in the unit-cell, k_B is Boltzmann's constant, and θ_D is a Debye temperature, corresponding to a high-frequency cut-off in the phonon density of states.

The thermal expansion of α-ammonium carbamate can be modelled reasonably well by use of a second-order Grüneisen-Debye model with a single Debye temperature. The first-order approximation shows a poorer fit to the data, even though in this case the first-order value of K_0 is marginally better than the second-order value, due to the first-order approximation [equation $(Eq. S1)(Eq. S1)(Eq. S2)$] being considered to be most valid at low temperature where the change in V is small. The better fit to the data of the second-order model comes at the expense of giving a bulk modulus value further away from that calculated by fitting the equation of state to the DFT simulations.

In previous studies (e.g. (Vočadlo *et al.*, 2002, Wood *et al.*, 2002, Hunt *et al.*, 2017)), it has been found that this Grüneisen-Debye model, or similar expressions taken to higher order, is capable of accurately modelling the thermal expansion of simple inorganic solids over a wide temperature range, thereby providing a method of estimating both their Debye temperatures and their elastic parameters. However, in the case of molecular solids, the presence of both inter- and intramolecular vibrations means that a more realistic calculation of the internal

energy, for example, using a double-Debye model fitted to experimental heat capacity data may be required if physically sensible elastic parameters are to be extracted from V(T) data. To obtain sensible values of the bulk modulus, for α-ammonium carbamate requires the use of models with at least two Debye temperatures; in this case, we adopt a model with two adjustable Debye temperatures:

$$U(T) = 9nk_B\left[Xf\left(\frac{\theta_{D1}}{T}\right) + Yf\left(\frac{\theta_{D2}}{T}\right)\right] \qquad (Eq.S4)$$

where $f(\theta_{D1}/T)$ and $f(\theta_{D2}/T)$ represent two distinct vibrational contributions to the internal energy of the crystal with characteristic high-frequency cut-offs defined by two Debye temperatures θ_{D1} and θ_{D2}. The magnitude of each of the relative functions is given by the mixing parameters X and Y.

However, a lack of heat capacity measurements of α-ammonium carbamate means a double-Debye model [equation $(Eq.S4)$], with two characteristic θ_D values, has significant estimated uncertainties on some of the fitted values, even though the fit to the data is excellent.

In the absence of heat capacity data, it is difficult to obtain precise values for both the Debye temperatures and the mixing parameters since these are poorly determined from thermal expansion data. It was necessary in this case to fix the value for the bulk modulus to that derived from fitting an equation of state to the E(V) points obtained in the *ab initio* simulations. The overall fit to the data very good, but the estimated uncertainties on the fitted Debye temperatures are quite large. The values of θ_{D1} and θ_{D2} are equivalent to vibrational frequencies of 172 cm^{-1} and 344 cm^{-1} respectively. The mixing parameters suggest that the heat capacity does not approach the classical high temperature limit of Dulong and Petit ($3Nk_B$) but instead tends to $2Nk_B$, with $X + Y = 0.37$, probably reflecting that ammonium carbamate cannot reach its high-temperature limit by virtue of decomposing at low temperature (below 273 K).

Table S4

Comparison of the parameters obtained by fitting Grüneisen-Debye models with a single Debye temperature and with two Debye temperatures to the unit-cell volume of α-ammonium carbamate between 4.2 K and 180 K.

	V_0 (Å)	K_0/γ (GPa)	θ_{D1} (K)	θ_{D2} (K)	X	Y	SSR
Single first-order	733.19(2)	40.1(4)	247(4)	-	1	-	271.68778
Double-Debye	733.14(1)	16 §	186(17)	495(95)	0.21(4)	0.16(3)	8.5E-5

§ Parameter is fixed during fitting to the value obtained by fitting the BMEOS3 to the E(V) points

S4. Fitting equations of state to the DFT simulations of α-ammonium carbamate and β-ammonium carbamate

An equation of state was used for fitting the E(V) and P(V) data to quantify thermodynamic parameters for each phase. A third-order Birch Murnaghan equation of state (BMEOS3, [21]) can be fitted to P(V) points via:

$$P(V) = \frac{3}{2}K_0\left(x^{\frac{7}{3}} - x^{\frac{5}{3}}\right)\left[1 + \frac{3}{4}(K_0' - 4)\left(x^{\frac{2}{3}} - 1\right)\right] \qquad (Eq.S5)$$

where x = V0/V. An integrated form of this equation can be fitted to an E(V) curve:

$$E(V) = \frac{9}{4}K_0V_0(x)\left[\frac{1}{2}(x)^{\frac{1}{3}} - \left(\frac{1}{x}\right)^{\frac{1}{3}}\right] + \left(\frac{9}{16}\right)K_0(K_0' - 4)V_0(x)\left[x - 3(x)^{\frac{1}{3}} + 3\left(\frac{1}{x}\right)^{\frac{1}{3}}\right] + E_0$$
$$- \left(\frac{9}{16}\right)K_0(K_0' - 6)V_0 \qquad (Eq.S6)$$
